# Role of Multivalency and Antigenic Threshold in Generating Protective Antibody Responses

**DOI:** 10.3389/fimmu.2019.00956

**Published:** 2019-05-01

**Authors:** Mark K. Slifka, Ian J. Amanna

**Affiliations:** ^1^Division of Neuroscience, Oregon National Primate Research Center, Oregon Health & Science University, Beaverton, OR, United States; ^2^Najít Technologies, Inc., Beaverton, OR, United States

**Keywords:** vaccines, antibody, immunological memory, VLP, HPV, JEV, HAV

## Abstract

Vaccines play a vital role in protecting our communities against infectious disease. Unfortunately, some vaccines provide only partial protection or in some cases vaccine-mediated immunity may wane rapidly, resulting in either increased susceptibility to that disease or a requirement for more booster vaccinations in order to maintain immunity above a protective level. The durability of antibody responses after infection or vaccination appears to be intrinsically determined by the structural biology of the antigen, with multivalent protein antigens often providing more long-lived immunity than monovalent antigens. This forms the basis for the Imprinted Lifespan model describing the differential survival of long-lived plasma cell populations. There are, however, exceptions to this rule with examples of highly attenuated live virus vaccines that are rapidly cleared and elicit only short-lived immunity despite the expression of multivalent surface epitopes. These exceptions have led to the concept that multivalency alone may not reliably determine the duration of protective humoral immune responses unless a minimum number of long-lived plasma cells are generated by reaching an appropriate antigenic threshold of B cell stimulation. Examples of long-term and in some cases, potentially lifelong antibody responses following immunization against human papilloma virus (HPV), Japanese encephalitis virus (JEV), Hepatitis B virus (HBV), and Hepatitis A virus (HAV) provide several lessons in understanding durable serological memory in human subjects. Moreover, studies involving influenza vaccination provide the unique opportunity to compare the durability of hemagglutinin (HA)-specific antibody titers mounted in response to antigenically repetitive whole virus (i.e., multivalent HA), or detergent-disrupted “split” virus, in comparison to the long-term immune responses induced by natural influenza infection. Here, we discuss the underlying mechanisms that may be associated with the induction of protective immunity by long-lived plasma cells and their importance in future vaccine design.

## Introduction

During an antigenic insult triggered by vaccination or infection, a series of immunological events unfold including the activation, proliferation, differentiation, and coordination of antigen-specific B cells and T cells that, if successful, will develop into an appropriate immune response that provides protective immunity upon re-encounter with the pathogen of interest. Pre-existing antibodies maintained in the circulation or along mucosal surfaces represent the first line of defense against reinfection and a better understanding of the parameters associated with inducing long-term antibody responses is key to the development of better, more effective vaccines.

## Role of Plasma Cells and Memory b Cells in Maintaining Long-Term Antibody Responses

Humoral immunity is built upon two main antigen-specific B cell populations; memory B cells (MBC) and plasma cells (PC). Although detailed discussion of MBC subsets and differentiation pathways are beyond the scope of this review, the reader is referred to several excellent reviews on these topics (1–3). In a broad sense, MBC and PC populations are often polar opposites in terms of basic phenotype and function. Class-switched MBC do not secrete antibodies *per se* but instead maintain expression of membrane-bound immunoglobulin that assists in their immunosurveillance capacity and increases their ability to bind and present antigen to CD4^+^ T cells through MHC Class II (4, 5). Upon activation, MBC are able to proliferate and differentiate into antibody-secreting plasmablasts/PC as well as generating more MBC. In contrast, long-lived PC are defined as terminally-differentiated non-dividing cells that no longer express membrane-bound immunoglobulins due to constitutive secretion of antibody molecules (6, 7). Fully differentiated PC also down-regulate MHC Class II expression (6, 7), resulting in a reduced ability to present peptide epitopes to CD4^+^ T cells. Early immunological studies indicated that PC (and most likely plasmablasts) had a relatively short lifespan, on the order of a few days to a few weeks (8–11). This finding was in stark contrast to the long-lived serum antibody responses that are commonly found after infection or vaccination and this led to several MBC-dependent theories for maintaining long-term antibody production, mostly involving reactivation and proliferation of MBC in order to repopulate short-lived plasma cell numbers. These theories have been described previously in detail (12–15) and questions regarding the need for MBC to replenish long-lived PC populations have been largely resolved based on analysis of PC survival after B cell depletion studies performed in mice (6, 16–18), humans (19–26), and non-human primates (NHP) (27). In addition, a recent case study using high-throughput sequence analysis of bone marrow-resident PCs from human subjects identified the persistence of multiple bone marrow PC clonotypes over a 6.5-year period, further supporting the concept of intrinsic PC longevity (28). Mice are a well-characterized model for studying humoral immune responses, but since mice live only 1–3 years, it is difficult to determine the true long-term survival of PC in the absence of MBC. Clinical studies in humans provide insight into the long-term durability of antibody responses after B cell depletion, but since CD20^+^ B cell depletion by rituximab may not fully deplete B cells from all lymphoid tissues (29–34), one could argue that a small proportion of residual MBC may still contribute to the maintenance of the PC pool. B cell depletion experiments performed in NHP provides another useful approach by combining the attributes of a versatile animal model with the ability to measure long-term antibody responses that can be followed for many years similar to those achieved in clinical studies. In longitudinal experiments spanning ~10 years of study, we monitored antiviral antibody responses to adenovirus, rhesus cytomegalovirus (RhCMV), and a measles-like paramyxovirus in addition to direct vaccine-mediated antibody responses to tetanus and three pertussis antigens including pertussis toxin, filamentous hemagglutinin (FHA), and pertactin (27). To determine the role of MBC in maintaining serum antibody levels, a group of animals had their peripheral MBC removed by anti-CD20 depletion in addition to surgical removal of the spleen and draining lymph nodes to eliminate the possibility of residual MBC populations or persisting antigen in the form of immune complexes on follicular dendritic cells (FDCs) residing in these lymphoid sites. Serum antibody responses to the aforementioned virus and vaccine antigens were monitored for several years and antibody titers in the MBC-depleted animals remained as durable as those observed in the non-depleted control animals. This indicates that MBC are not required to maintain antigen-specific PC populations and long-term antibody production. As a secondary approach to identifying long-lived PC, BrdU (bromodeoxyuridine) incorporation studies were performed at the time of vaccination and non-dividing BrdU^+^ PC could be detected by histology for up to 10 years after immunization/BrdU administration. Together, these independent approaches provide further support for the theory that non-dividing PC can persist for many years and continue to maintain serum antibody levels in the absence of an intact MBC pool.

It is important to note that although MBC are not required for directly maintaining PC numbers and serum antibody titers, they still play an important role in host defense (3). In cases in which pre-existing antibody responses are too low to block infection, MBC may traffic to sites of infection as well as contribute to rapid anamnestic antibody responses. Another interesting attribute of MBC is that they have been found to develop a broader range of specificities than those observed in the primary PC pool and have the capacity to provide protection against variants of the original pathogen that escape neutralization by immunodominant serum antibodies (35). For some viruses such as hepatitis B virus (HBV), MBC may contribute directly to protection since cases have been reported of vaccinated individuals who are at least transiently infected with HBV and seroconvert to non-structural HBV antigens but do not develop into a carrier state or show clinical manifestations of disease (36, 37). This indicates that for pathogens that are slow at inducing a disease state, there may be enough time for anamnestic MBC-mediated immune responses to participate in clinically relevant immune defense.

## Multivalent Whole-Virus or Virus-Like Particle (VLP) Vaccines Induce Long-Term Immunity

One commonly held misconception regarding immunological memory is the idea that live-attenuated vaccines elicit life-long immunity after a single immunization whereas non-replicating protein vaccines elicit only short-lived immunity (38). This assertion most likely comes from prior clinical observations in which natural infection in childhood often leads to lifelong immunity but when vaccines against those same pathogens are developed using inactivated or live-attenuated strains of the wild-type pathogen, in many cases a booster vaccination is required in order to sustain host immunity above a protective threshold (38). Here, we provide four examples of multivalent VLP or whole-virus vaccines that elicit long-term, potentially life-long immunity after completion of the recommended vaccination series.

**Figure 1:**
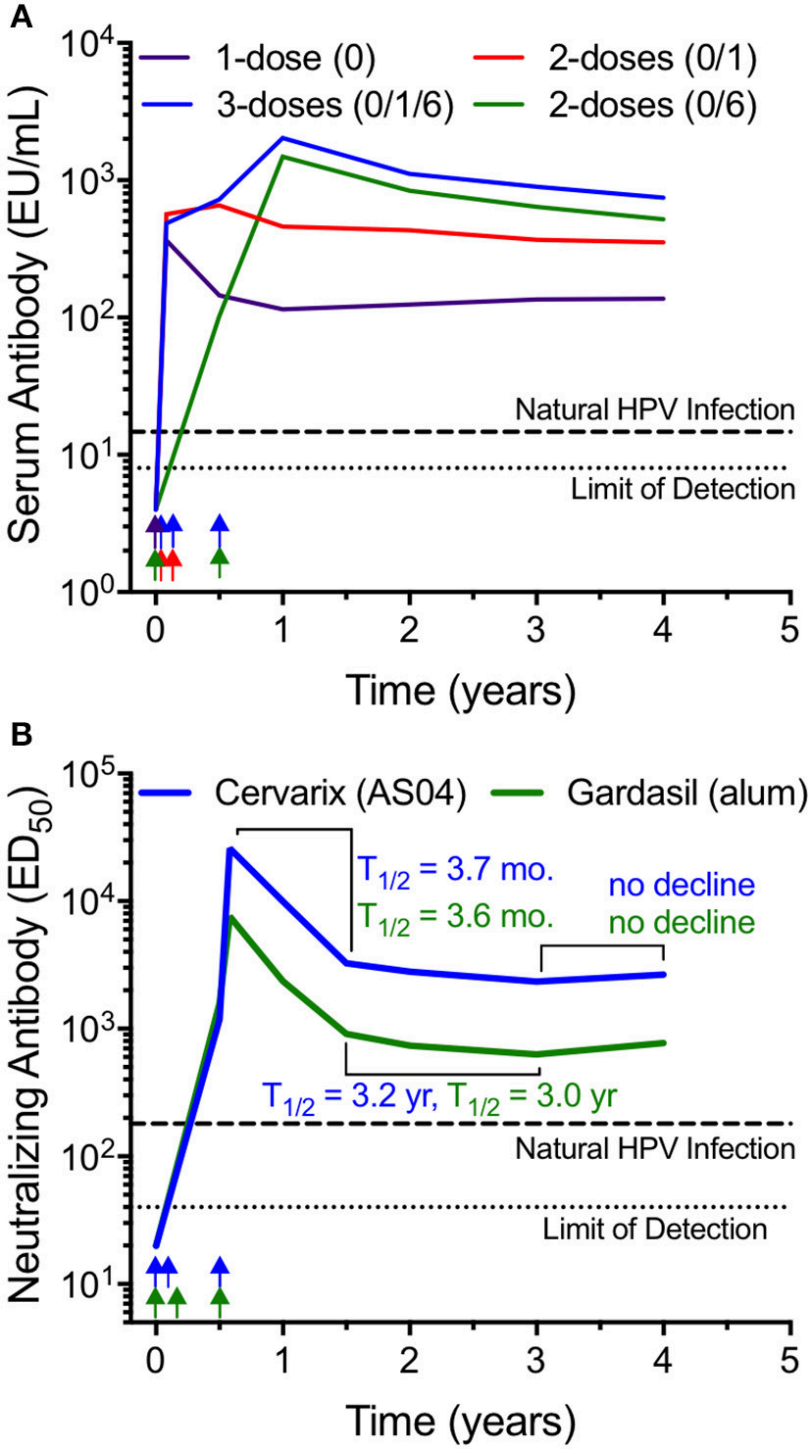
Long-term antibody maintenance after vaccination with HPV VLP vaccines with or without Toll-like receptor 4 (TLR4) agonists. **(A)** Serum antibody responses to HPV16 were monitored by ELISA for up to 4 years following various vaccination schedules (1 dose; 2 doses at 0 and 1 month; 2 doses at 0 and 6 months; 3 doses at 0, 1, and 6 months) with AS04-adjuvanted, VLP-based Cervarix® HPV vaccine. The graph was prepared using data provided in Table 1 from Safaeian et al. (42). Longitudinal analysis was limited to subjects with sera available at each time point, with group sizes ranging from 52 to 140. **(B)** Neutralizing antibody responses against HPV16 were assessed longitudinally following a three-dose vaccination schedule with either Cervarix (AS04 adjuvant), or Gardasil® (alum-only adjuvant). The graph was prepared by combining the HPV16-specific neutralizing antibody titers from 3 age groups (18–26, 27–35, and 36–45 years of age) using the geometric mean of the data presented in Figure 2 from Einstein et al. (43). This dataset was comprised of 136 subjects that received Cervarix and 139 subjects that received Gardasil. For both panels, antibody levels after natural HPV infection are shown as a dashed line and the limit of detection is shown as a dotted line. Approximate vaccination time points are indicated by arrows. EU; ELISA Units, ED_50_; 50% effective dose.

Human papilloma virus (HPV) is a non-enveloped DNA virus that is 55 nm in diameter that causes cervical cancer as well as a number of other genital and oropharyngeal cancers. Globally, cervical cancer is the third leading cause of cancer among women with an estimated 530,000 new cases and 275,000 annual deaths (39). There are currently three HPV vaccines on the market; Cervarix (HPV16/18), Gardasil (HPV6/11/16/18), and Gardasil-9 (HPV6/11/16/18/31/33/45/52/58). Each vaccine is formulated with alum except that Cervarix contains AS04, a combination of alum and the toll-like receptor 4 (TLR4) agonist, monophosphoryl lipid A (MPL). Each vaccine is composed of VLP comprising an array of 360 copies of the L1 major capsid protein arranged in a densely packed pentameric array (40). HPV vaccination was initially designed as a 3-dose vaccination series. However, in a Phase III trial of Cervarix in Costa Rica, researchers were surprised to find that 4-year vaccine efficacy against persistent HPV16/18 infection was similarly high among women who received 1, 2, or 3 doses of vaccine (41). Further analysis of serum antibody titers provided a unique opportunity to determine the kinetics of antibody decay in relation to different booster vaccination regimens (Figure 1A) (42). Following the 3-dose regimen, high titers of anti-HPV16 (and anti-HPV18) were maintained for the 4-year span of observation. Similar results showing a well-maintained plateau of antibody production have been observed in prior studies with clinical observations of stable protection against disease and stable antibody titers for up to 9 years after vaccination (39). Likewise, long-term antibody responses have also been observed after Gardasil-9 vaccination, indicating that even when 9 closely related VLP antigens are combined into one vaccine, long-term immunity to each of the individual virus serotypes is maintained (44). We calculated that after an initial antibody decay rate of T_1/2_ = 14 months from years 1 to 2 after a 3-dose regimen in the Costa Rican study, the antibody half-life from years 2 to 4 post-vaccination slows to ~T_1/2_ = 3.5 years (Figure 1A). Following a 2-dose schedule (either 0/1 month or 0/6 month regimen), the initial antibody decay rates appeared to be similar to the 3-dose series (T_1/2_ = 11.6 and 14 months for the 0/1 and 0/6 schedules, respectively) and between years 2 and 4, the antibody T_1/2_ = 2.9–6.7 years. Although the 0/6 vaccination group achieved higher peak antibody titers compared to the 0/1 group, by 4 years post-vaccination the antibody titers appeared similar. These results may be unique to HPV since booster immunization studies with other vaccines indicate that longer time intervals between booster vaccinations often results not only in higher peak titers, but also appears to increase levels of antibodies during the early plateau phase of the vaccine-mediated immune response (37, 45). Importantly, the peak and plateau levels of anti-HPV antibodies are similar following the 2-dose and 3-dose schedules and based on the information provided to the ACIP (Advisory Committee on Immunization Practices) showing that a) the 3-dose schedule elicits long-term protection and b) a 2-dose schedule elicits similar antibody titers to a 3-dose schedule, in 2016 the ACIP changed their HPV vaccine recommendations from a 3-dose to a 2-dose schedule for girls and boys ages 9 through 14 years (46).

Interestingly, following a single HPV vaccination the initial antibody decay rate from 1 to 12 months was T_1/2_ = 6.7 months but showed no appreciable decline from 2 to 4 years post-vaccination. The single dose vaccination schedule is noteworthy because although antibody titers plateaued at levels that were about 5-fold lower than that achieved by the 2-dose or 3-dose series, these antibody titers are still maintained above the levels observed after natural infection and it shows that a booster vaccination (or the accompanying re-activation of MBC during a boosted immune response) is not required in order to generate long-lived PC that can maintain protective steady-state antibody production. This also indicates that HPV vaccination may be one of the few vaccines capable of inducing prolonged, potentially life-long immunity after a single vaccination. However, this may pertain only to protection against vaccine-targeted serotypes HPV16 and HPV18 since further analysis indicates that a single dose of vaccine was suboptimal compared to the 3-dose series at inducing cross-reactive antibody responses to other clinically relevant HPV serotypes including HPV31 and HPV45 (47). This suggests that similar to other vaccines (38), even the impressive immunological memory induced by HPV vaccination might be further improved by administering at least one booster vaccination.

The role of TLR agonists in generating durable antibody responses is a question of considerable interest and much has been learned by studies involving HPV vaccination. Unlike inactivated whole virus particles that may contain viral RNA with the potential to trigger TLR7 activation, HPV VLP do not contain viral RNA yet still induce long-lived, potentially lifelong antibody responses (Figure 1). During clinical development of Cervarix, HPV16/HPV18 VLP vaccine antigen was prepared either with AS04 (i.e., alum plus MPL) or with alum alone to assess the role of TLR4 engagement by MPL on vaccine-induced antibody responses during HPV vaccination (48). Peak anti-HPV titers occurred at 1 month after the third dose of vaccine and were ~2- to 3-fold higher among the subjects immunized with the MPL-containing AS04 formulation compared to vaccination with HPV containing only the alum adjuvant regardless of whether HPV-specific ELISA or pseudo-neutralizing assays were employed (48). However, by 3.5 years after the last administered dose of vaccine, there was only a 1.5-fold (HPV16) to 2.1-fold (HPV18) difference in virus-specific antibody titers, indicating that although TLR4 stimulation may increase peak antibody levels, the difference in the magnitude of antibody responses becomes relatively minor within the first few years after vaccination.

Another longitudinal study comparing Cervarix (AS04) to Gardasil (alum-only) provides further insight into the role of TLR stimulation for inducing long-lived antibody responses (Figure 1B) (43). Following each 3-dose vaccination series, anti-HPV antibody titers peak at 7 months (i.e., 1 month after the final vaccination) and decay with similar initial half-life estimates of T_1/2_ = 3.7 and 3.6 months for Cervarix and Gardasil, respectively. From 1.5 to 3 years post-primary vaccination (i.e., 1–2.5 years post-final vaccination), antiviral antibodies decay with slower, albeit similar kinetics with half-life estimates of T_1/2_ = 3.2 and 3.0 years, respectively. By 3–4 years post-primary vaccination, anti-HPV antibody titers have plateaued, with no further evidence of decline following administration of either vaccine formulation. Together, these studies demonstrate that a standard alum formulation provides durable antiviral immunity and addition of a TLR4 agonist appears to play little to no role in the overall durability of long-lived vaccine-mediated antibody responses.

**Figure 2:**
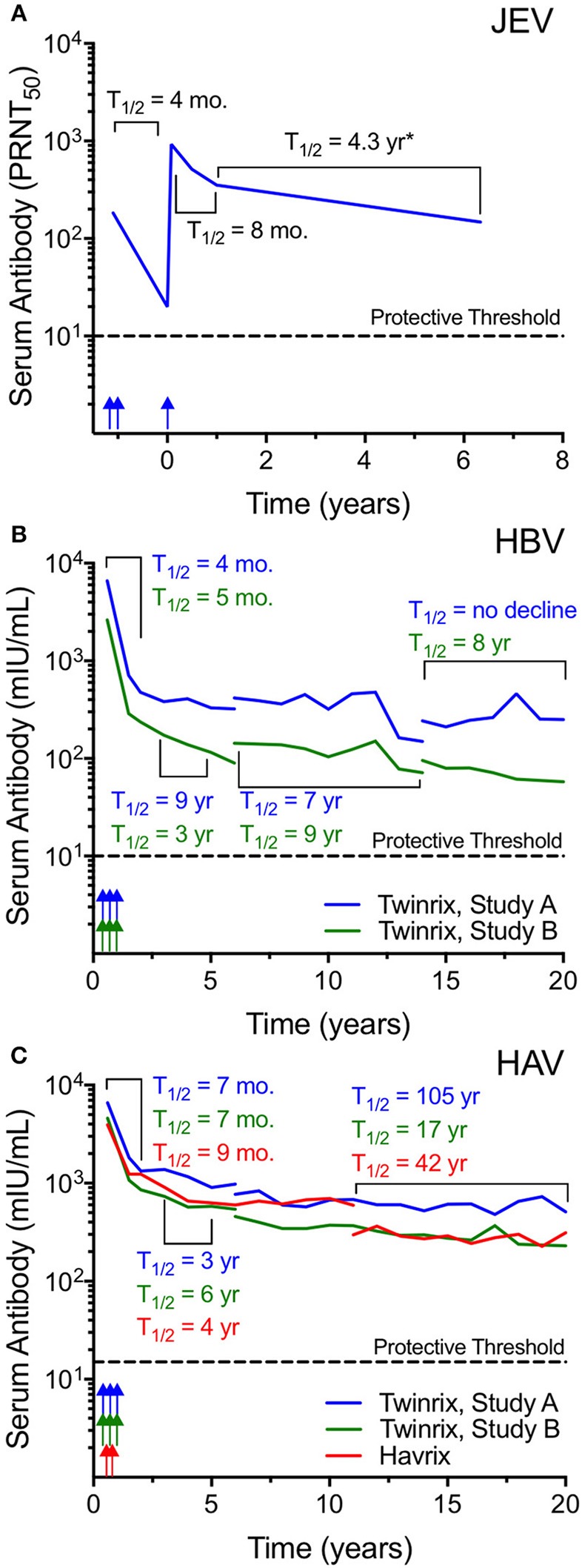
Inactivated whole-virus vaccines induce long-lived antibody responses. **(A)** The alum-adjuvanted, inactivated whole-virus Ixiario® JEV vaccine was used to immunize 67 subjects with a two-dose primary series spaced 28 days apart followed by a third dose administered at 15 months after the conclusion of the primary series. The figure was constructed using the primary data provided in Table 2 of Paulke-Korinek et al. (53). Serum samples were assessed for JEV-specific neutralizing activity (plaque-reduction neutralizing titer 50%; PRNT_50_) for up to 6 years post-vaccination. The putative protective threshold of PRNT_50_ ≥ 10 is shown as a dashed line. Vaccination time points are indicated by arrows. **(B,C)** Vaccine-induced serum antibody responses were monitored for up to 20 years in subjects receiving either a combined hepatitis B virus (HBV) and hepatitis A virus (HAV) vaccine (Twinrix®), or an HAV-only vaccine (Havrix®) (54, 55). HBV surface antigen (HBsAg) forms pleomorphic, irregular-shaped VLP whereas HAV consists of purified, inactivated whole-virus and both vaccines are formulated with alum. The Twinrix vaccine **(B,C)** was administered as a three-dose series (0, 1, and 6 months) and included 2 cohorts (Study A and Study B). Subject attrition resulted in fewer subjects included at later time points with 18/107 subjects in Study A and 22/116 subjects in Study B completing visits up to 20 years post-vaccination. The Havrix vaccine (**C**) was administered as a two-dose series at 0 and 6 months (55) with 34/76 subjects completing the 20 year study. Color-coded arrows indicate the approximate vaccination schedule. At various points, assay kits for HBV and HAV antibody measurements changed, and these changes are indicated by line breaks. Data for geometric mean antibody titers were obtained from clinical study reports available on-line (https://gsk-clinicalstudyregister.com/) and antibody half-lives were calculated for the select periods as indicated. The putative protective threshold for HBV and HAV are indicated with dashed lines. mIU/mL; milli-international units per milliliter. ^*^JEV-specific antibody half-life may underestimate the durability of antibody responses after vaccination due to inclusion of the 1-year time point, a period in which antibody responses have not plateaued and are still declining rapidly.

Japanese encephalitis virus (JEV) is an enveloped flavivirus that is endemic in South-East Asia and the Western Pacific. The JEV vaccine, IXIARO, is FDA-approved for use in the U.S. for the purposes of protecting international travelers and military personnel who plan on entering JEV-endemic areas. The vaccine contains alum-adjuvanted formalin-inactivated whole virus that is ~50 nm in diameter. Flaviviruses are secreted from infected cells as a mixture of mature and immature virus particles. In its immature form, flavivirus particles contain 60 heterotrimers of the envelope and premembrane protein arranged in an icosahedral configuration (49, 50) whereas in its mature form, the virus is comprised of 180 copies of the envelope protein in 90 antiparallel dimers arrayed in a herringbone fashion (51, 52). Following a primary JEV vaccination series of doses administered at 0 and 1 month, the geometric mean titers (GMT) reached 1:183 by 1 month after the second dose (Figure 2A) (53). Antibody titers declined rapidly (T_1/2_ = 4 months) during the first year after vaccination to a GMT of 1:20 and even though 96% of subjects were still seropositive at this time point, this represents a neutralizing titer that is only 2-fold above the protective threshold of 1:10. A third dose of vaccine resulted in much higher antibody titers that peaked at 1:927 by day 28 after the third dose and this elicited long-term immunity in which 96% of subjects remained seropositive (GMT = 1:148) when examined at 6 years after final vaccination (Figure 2A). The authors used mathematical modeling to estimate the duration of protection using a log linear function with a structural break at 6 months post-booster vaccination and predicted that protection would last 14 years. When we estimated antibody half-life based on this dataset there was approximately an 8 month half-life from the peak through the first year after the third dose and from 1 to 6 years after booster vaccination the average antibody T_1/2_ = 4.3 years. However, these estimates are likely to be an underestimation of the true durability of the antiviral immune response because they are based in part on the 1 year time point when antibody responses are still rapidly declining and based on the data in Figures 1, 2, and other studies (14, 56–58), antibody decay rates typically do not reach their stable kinetic profiles until 3–4 years after vaccination or infection. This means that the >90% seroprotection rate observed at 6 years after booster vaccination may represent a plateau that could be maintained for much longer than the estimated 14-year time frame. Future studies will require obtaining samples during the plateau phase of the immune response between 3 and 10 years post-vaccination in order to accurately predict the persistence of immunity at later time points but it is possible that a 3-dose regimen could provide long-term and potentially lifelong protection against JEV.

Hepatitis B virus (HBV) is a major cause of human morbidity and mortality with an estimated 620,000 HBV-associated deaths occurring globally in 2000 and implementation of routine infant immunization is anticipated to reduce of HBV-associated fatalities by >80% (59). The native HBV particle is ~42 nm in diameter whereas the HBV vaccine contains subviral particles comprised of hepatitis B surface antigen (HBsAg) that are ~22 nm in size (60, 61). *In vivo*, the HBV HBsAg is produced in vast excess as a decoy molecule and it is pleomorphic with shapes that range from spherical or ovoid to rod-shaped, tubular or even filamentous (37). There are several recombinant HBV VLP vaccines on the market with at least 25 vaccines that have been pre-qualified by the World Health Organization (37). The Twinrix^TM^ vaccine is a combination vaccine against HBV and hepatitis A virus (HAV) that is formulated with alum and the duration of vaccine-mediated antibody responses to Twinrix have been monitored for 20 years among two cohorts; Study A and Study B (Figures 2B,C) (54). Following a 3-dose schedule administered at 0, 1, and 6 months, anti-HBV antibody responses decline rapidly for the first 2 years after vaccination (T_1/2_ = 4–5 months) followed by more durable maintenance of antibody levels thereafter (Figure 2B). Over the 20 years of observation, there were several technical changes that resulted in breaks in the longitudinal studies when switching from one serological assay to another. From 3 to 5 years post-vaccination, antibody T_1/2_ = 3–9 years, from 6 to 14 years post-vaccination, HBV-specific antibody T_1/2_ = 7–9 years, and from 14 to 20 years after vaccination, anti-HBV titers continued to decline with T_1/2_ = 8 years to infinity (i.e., no decline observed in Study A). The apparent increase in antibody levels near the end of Study A appears to be an aberration when compared to the earlier time points within this cohort as well as to the decay rates observed among Study B subjects. The increase in antibody from 15 to 20 years post-vaccination may be due to exposure event(s) or possibly due to the limitations associated with study subject attrition with fewer individuals included at these later time points [at Year 20 there were 28/150 subjects (18.6%) in Study A and 25/131 (19%) subjects remaining in Study B]. Mathematical modeling predicted that ≥50% of vaccinated subjects would maintain antiviral antibodies at ≥10 mIU/mL for 40 years after vaccination (54). The decline in antibody titers below this threshold in itself may not be of great concern because although it is recognized that serological titers of ≥10 mIU/mL protect against HBV infection, as long as subjects have received at least 3 doses of an HBV vaccine and developed an initial antibody response, they continue to be protected against chronic HBV infection and clinical disease (36, 37).

Hepatitis A virus (HAV) is endemic in many low- and middle-income countries with millions of people at risk and it is associated with ~15,000 deaths annually (62). HAV is a non-enveloped icosahedral virus particle that is ~27 nm in diameter (63–66) and comprised of 32 capsomeres (each capsomere containing 5 protomers with each protomer made of 3 proteins; VP1, VP2, and VP3). There are a number of inactivated HAV vaccines on the market including Twinrix, Havrix, Avaxim, Epaxal, Healive, and VAQTA. The durability of HAV vaccination (Havrix) following a 2-dose schedule (55) was compared to the HAV titers elicited after the 3-dose Twinrix vaccination series containing both HAV and HBV antigens (Figure 2C). Similar to HBV (Figure 2B), anti-HAV antibody titers declined rapidly during the first 2 years after vaccination with T_1/2_ = 7–9 months. This was followed by a slower antibody decay rate of T_1/2_ = 3–6 years from 3 to 5 years after vaccination. There was more variability at the later time points measured from 11 to 20 years post-vaccination with antibody T_1/2_ = 105, 17, and 42 years for Twinrix Study A, Twinrix Study B, and Havrix, respectively. Similar to HBV, the increased variability in half-life estimates is likely due to the attrition of participating study subjects over time but nevertheless the data shows that antibody responses are durably maintained well-above the protective threshold. Moreover, this data indicates that there is no further benefit to a 3-dose schedule over a 2-dose schedule (Twinrix vs. Havrix) in terms of the induction of long-term and potentially life-long immunity against HAV. Mathematical modeling studies of anti-HAV antibody responses indicate that they are likely to persist for 25 years, 40 years or even longer (55, 67, 68) without requiring further booster vaccination in order to maintain long-lived PC numbers and protective serum antibody levels.

Review of the vaccine-induced antibody responses to the 4 representative viruses described in Figures 1, **2** show several similarities as well as some interesting differences. In terms of similarities, the early kinetics of antibody decay during the first 1–2 years after booster vaccination declined with a 4- to 14-month half-life for each of the vaccines. From 2 to 4 years post-booster vaccination (HPV), 3–5 years post-booster vaccination (HBV, HAV), or 1–6 years post-booster vaccination (JEV), antibody responses declined with approximately a 3- to 9-year half-life regardless of the vaccine antigen under study. This indicates that for alum-adjuvanted protein vaccines, there may be a relatively common set of early decay rate curves that could potentially be used to predict long-term antibody maintenance. In contrast, at >5 years post-vaccination, comparison of HBV and HAV titers revealed a different story. Although their first two stages of decay rate kinetics were similar, at later time points the HBV titers continued to decline with a 7–9-year half-life similar to that observed from samples taken 3–5 years post-vaccination whereas the HAV titers had stabilized with a 17-, 42-, or 105-year half-life (roughly, average half-life of 55 years). These differences are unlikely to be due to variability in vaccination procedures since the Twinrix studies included both HBV and HAV antigens within the same vaccine formulation. Another proposed reason for the more rapid antibody decay rate kinetics observed with HBV vaccination is that the HBV subviral particle/VLP is a relatively small particle (~23 nm) compared to other VLP such as HPV. However, the intact HAV virus particle is similar in size (~27 nm) and so it seems unlikely that particle size alone can explain the differences in long-term antibody maintenance between HAV and HBV obtained from the same vaccine (Twinrix). The 7–9-year half-life observed at late time points after HBV vaccination is reminiscent of other data with monovalent protein antigens such as tetanus and diphtheria, which have longitudinal half-lives of 11–19 years, respectively (58, 69). In contrast to the more rigid structure of the non-enveloped HAV particle, HBV HBsAg-based VLPs are unusual because they are about half of the size of the native infectious virus particle, contain small 1 nm pores that are thought to be channels for small molecules (70), and are pleomorphic with examples of different sized spherical, ovoid, or even filamentous forms observed *in vivo* (37, 70–72). During clinical vaccine production in yeast cells, more recent studies noted that by electron microscopy, HBV VLPs were 23.09 nm in diameter (range; 20–30 nm), but when the same vaccine material was measured by dynamic light scattering (DLS), the average particle size was 55.91 nm and the size distribution of HBV VLP ranged broadly from 20 to 180 nm (60). It is believed that larger particles were not observed because of a sterile filtration step during the manufacturing process that would have removed particles>200 nm in diameter. However, this indicates that in practice, the yeast-derived HBsAg/VLPs produced in clinical vaccine preparations may often be dimeric or constitute even larger agglomerates (confirmed by electron microscopy) and if these antigenic preparations are aggregates (60, 73) that have a disregular order, we speculate that multimeric crosslinking of the B cell receptor (BcR) on antigen-specific B cells could be suboptimal. This may explain why antiviral antibodies mounted against the HBsAg subviral particles have a half-life that is more similar to alum-adjuvanted non-repetitive proteins such as tetanus and diphtheria, rather than the more long-lived antibody responses induced by the highly repetitive structures on HAV particles within the same vaccine formulation (Figure 2C).

## Influenza: Comparison of Repetitive vs. Non-Repetitive Antigen

Despite more than 60 years of vaccine research and development, influenza remains a pressing public health concern, with the US experiencing >200,000 hospitalizations and over 20,000 deaths per year (74–76). The 2017–2018 season was particularly severe with ~900,000 influenza-associated hospitalizations and 80,000 influenza-associated deaths in the US (77). This indicates that despite vaccination coverage in 2017–2018 of up to 47% of the US population (and ~68% of children aged 6 months to 4 years) (77), more work is needed to find new and improved vaccines against this potentially life-threatening disease. Influenza A is responsible for the majority of human infections and while comprised of many different subtypes, it is H1N1, H2N2, and H3N2 that are the primary subtypes associated with pandemics and large seasonal outbreaks observed over the last 100 years (78, 79). Until recently, the durability of immune responses to influenza in the absence of reinfection were difficult to determine due to the potential for re-exposure to the same strains or similar antigenically-related “drifted” strains of the virus that caused the initial infection. However, a recent study in Finland describing the 2009 influenza A/H1N1 pandemic [A(H1N1)pdm09] has revealed evidence that subtype-specific immunity against influenza may indeed be lifelong. Using banked serum samples obtained in 2005 (i.e., prior to the pandemic of 2009), the study found that ≤ 3% of samples from subjects born between 1950 and 2005 had cross-reactive hemagglutinin inhibition (HAI) antibodies to A(H1N1)pdm09 (80). This indicated that cross-reactive H1N1 strains had not been circulating during this prolonged 55-year span of time. In contrast, 96% of subjects born between 1909 and 1919 had HAI titers of ≥1:10 against A(H1N1)pdm09 and this was likely due to maintenance of antiviral antibody titers for decades after recovery from the H1N1 1918 Spanish flu pandemic because of the structural and sequence similarities of the hemagglutinin (HA) surface antigen between the H1N1 1918 Spanish flu and the 2009 pandemic A(H1N1)pdm09 strain. Remarkably, ~55% of the 1909–1919 cohort still retained HAI titers of ≥1:40, suggesting that these were potentially seroprotective levels of antiviral antibodies and this could explain multiple epidemiological reports of pre-existing antibodies and reduced disease among individuals over 65 years of age during the 2009 pandemic (80). These results are not unique to the H1 influenza antigen since similar results were observed 77 years after an H3-like influenza pandemic in 1889-91 that, “.*.left a lifelong immunological imprint on* ≥*80% of those who were aged* ≤ *21 years at the time*” (81). Together, these results indicate that natural infection can elicit lifelong subtype-specific immunity against influenza. Although annual variations within a subtype (antigenic drift) as well as changes between subtypes over time (antigenic shift) have made influenza vaccine development challenging (82), there is concern that current influenza vaccines induce only transient immunity that may not be sufficiently maintained for the duration of a single season even in the absence of demonstrable antigenic drift (83). What could be some of the reasons for the disparity between lifelong immunity induced by natural influenza infection vs. rapidly waning immunity after influenza vaccination?

**Figure 3:**
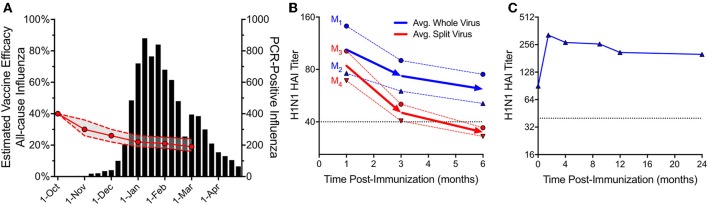
Protective immunity after vaccination with repetitive (whole-virus) or non-repetitive (split-virus) influenza vaccines. Current inactivated influenza vaccines contain detergent-disrupted or “split” virus antigens and these formulations lose protective efficacy rapidly during the course of a single season. **(A)** A study of 7 influenza seasons involving 44,959 patients showed that the odds ratio (OR) for testing positive with influenza virus increased by ~16% for each 28-day period after vaccination. If vaccine efficacy (VE) was 40% starting on October 1^st^, we estimate that the subsequent decline in VE would result in only 20% VE during the peak of an average influenza season. The VE and associated 95% confidence intervals were inferred from the ORs in Table 2 (VE = [1÷OR] × 40%) of Ray et al. (85). Data for total PCR-positive influenza tests from 2010/11 to 2016/17 seasons were adapted with permission from Figure 1 in Ray et al. (85). **(B)** In 1977, hemagglutination inhibition (HAI) responses to 2 commercial whole virus (WV) vaccines (dotted blue lines, M_1_, manufacturer 1; M_2_, manufacturer 2) and 2 split virus (SV) vaccines (dotted red lines, M_3_, manufacturer 3; M_4_, manufacturer 4) were monitored in parallel following a single immunization of adults (*n* = 46-56/group) (86). HAI serum titers were adapted with permission from Figure 1 in Cate et al. (86) with the average of each group (WV or SV) shown as solid lines. **(C)** In 1953, a single dose of WV vaccine was administered to 45 young adults and HAI titers were monitored longitudinally for up to 2 years after vaccination (87). Antibody titers against the PR8 (A/H1N1) strain of influenza were adapted with permission from Figure 1 in Salk et al. (87). For panels **(B)** and **(C)**, the putative protective HAI antibody level of 1:40 is shown as a dotted line.

Despite clear evidence that natural influenza can induce long-term immunity, recent studies have shown that the efficacy of current detergent-disrupted or “split” influenza vaccines may wane rapidly over the course of a single influenza season (84). One large study performed over the course of seven influenza seasons and involving 44,959 patients, found that the odds ratio (OR) for testing positive for any influenza increased by ~16% for each additional 4-week period following vaccination (85). The authors of this study assumed a peak vaccine efficacy (VE) of 40% on October 1^st^, and using this assumption along with the measured decreases in VE over time, these results indicate that protection could decline to as little as ~20% by the peak of an average influenza season (Figure 3A), leaving a substantial proportion of the population at risk. In a comparable report encompassing a nationwide influenza network involving five locations and four influenza seasons (2011/12–2014/15), US researchers demonstrated declining VE for influenza A (both H3N2 and H1N1pdm09) and influenza B, with rates in absolute VE dropping by ~7–11% per month (88). Declining immunity to H3N2 was particularly pronounced, dropping to a VE point estimate of 0% by ~5 months after vaccination. A similar multicenter trial performed among multiple European countries, spanning the 2010/11 through 2014/15 seasons, demonstrated peak VE of 50.6% for the H3N2 component at 38 days post-vaccination, which dropped to 0% VE by ~3 months (89). As noted above, one explanation for intra-season waning of protective immunity could be due to antigenic drift resulting in immunological escape of circulating influenza strains as the season progresses. However, several studies suggest that mechanisms distinct from antigenic mismatch may be contributing to waning vaccine efficacy (90, 91). In one study performed in the UK during the 2011/12 influenza season, protective immunity declined from 53% VE at < 3 months post-vaccination to only 12% VE at points ≥3 months post-vaccination (90). These differences in VE could not be attributed simply to antigenic drift because HA antigens for ≥78% of A/H3N2 strains isolated during early (Oct-Jan) or late (Feb-Apr) periods remained antigenically similar to the vaccine strain. This suggests that despite a nearly 5-fold drop in VE over the course of the season, the antigenic characteristics of circulating influenza strains remained relatively stable. In Spain during the 2011-2012 influenza season, VE efficacy waned rapidly over time with VE = 52%, 40%, and 22% at 3.5 months, 3.5–4 months, and >4 months, respectively after vaccination even though no changes in circulating virus strains were identified throughout the season (83). During the 2015/16 influenza season in Canada, early season (January-February) VE against A/H1N1pdm09 reached 62%, but dropped to a non-significant level of 19% during the March-April period of observation. This decline occurred even though only 1 of the 467 A/H1N1pdm09 viruses isolated during the season was considered antigenically distinct from the vaccine strain (91). Together, these independent studies demonstrate that regardless of antigenic drift, the failure of current detergent-split influenza vaccines to elicit durable protection is likely due to rapidly waning immunity after immunization.

Could changes in modern influenza vaccine manufacturing processes be playing a role in low overall immunogenicity and rapidly waning protective immunity? Although vaccine developers utilized inactivated whole virus (WV) influenza vaccines for several decades, concerns over reactogenicity led to the development of detergent-split virus (SV) vaccines (92). The first split vaccine was licensed in the US in 1968 to reduce reactogenicity observed with earlier WV formulations (93). Subsequently, a series of clinical trials comparing WV and SV formulations for the 1976 swine flu demonstrated higher reactogenicity of WV formulations in children and these results are credited with influencing later decisions to recommend using only SV-type influenza vaccines in this age group (92). At the time, industry leaders cautioned that preference from the medical community for SV formulations could lead to the eventual disappearance of WV vaccines (94). This was a concern because SV vaccines are poorly immunogenic compared to their WV counterparts. For example, children vaccinated with WV vaccines showed fourfold or greater rise in antibody responses in 69-100% of subjects whereas those vaccinated with SV formulations produced fourfold responses in 0-23% of the vaccine recipients (94, 95). Likewise, 65–93% of 3–5-year old children receiving WV formulations demonstrated HAI titers of ≥1:20 whereas 0–10% of children receiving SV preparations mounted HAI responses of ≥1:20 (94). The concerns over vaccine reactogenicity outweighed the merits of immunogenicity and Maurice Hilleman noted at the time that, “*The production of vaccines in this country is very closely geared to consideration of costs for preparation and to preferences in the medical community. Responsible manufacturers are sensitive to these demands and can and will produce whatever kind of vaccine is wanted*” (94). In addition to issues of poor seroconversion rates among young and naïve vaccine recipients, another potential concern with SV vaccine preparations involved the durability of vaccine-induce immune responses. Contemporary comparisons of vaccine-mediated antibody persistence between matched WV and SV influenza formulations are scarce. However, during the clinical testing of the 1976 A/H1N1 swine flu vaccine, groups of seronegative adults received one of four different commercially manufactured vaccines including two WV vaccines or two SV vaccines and antibody titers were monitored for up to 6 months after immunization (Figure 3B) (86). Titers declined rapidly from 1 to 3 months post-immunization with slower decay rate kinetics observed from 3 to 6 months post-vaccination. However, the authors noted a significant difference (*P* < 0.05) in the rate of antibody decline between the two vaccine types, with WV vaccines declining by 40% over a 5-month period, compared to an average decrease of nearly 60% for SV vaccines, leading to an improvement in serum HAI titers for the WV vaccines by the end of the observation period. These differences become important as antibody titers decline to near a putative protective threshold such as an HAI titer of 1:40. The observation that WV vaccines elicit antiviral immunity that was on average still maintained above 1:40 at 6 months post-vaccination whereas SV vaccines induced antibody responses that declined to < 1:40 may explain the epidemiological findings of short-lived protective immunity in our current populations during this short period of time after vaccination (Figure 3A). In contrast to SV vaccines, it is possible that WV vaccines will induce more durable immunity and in a separate study in which HAI titers were monitored for up to 2 years following WV influenza vaccination in pre-immune subjects (87), the HAI titers peaked at 6 weeks post-vaccination, declined during the first year after vaccination, but then remained relatively stable for at least 2 years above the protective threshold (Figure 3C). This work suggests that re-introduction of optimized, multivalent WV influenza vaccine formulations may provide a feasible approach to improving the durability of current seasonal influenza vaccine regimens if issues surrounding potential reactogenicity are addressed.

## Role of Multivalency and Antigenic Threshold in Determining Duration of Protective Immunity

**Figure 4:**
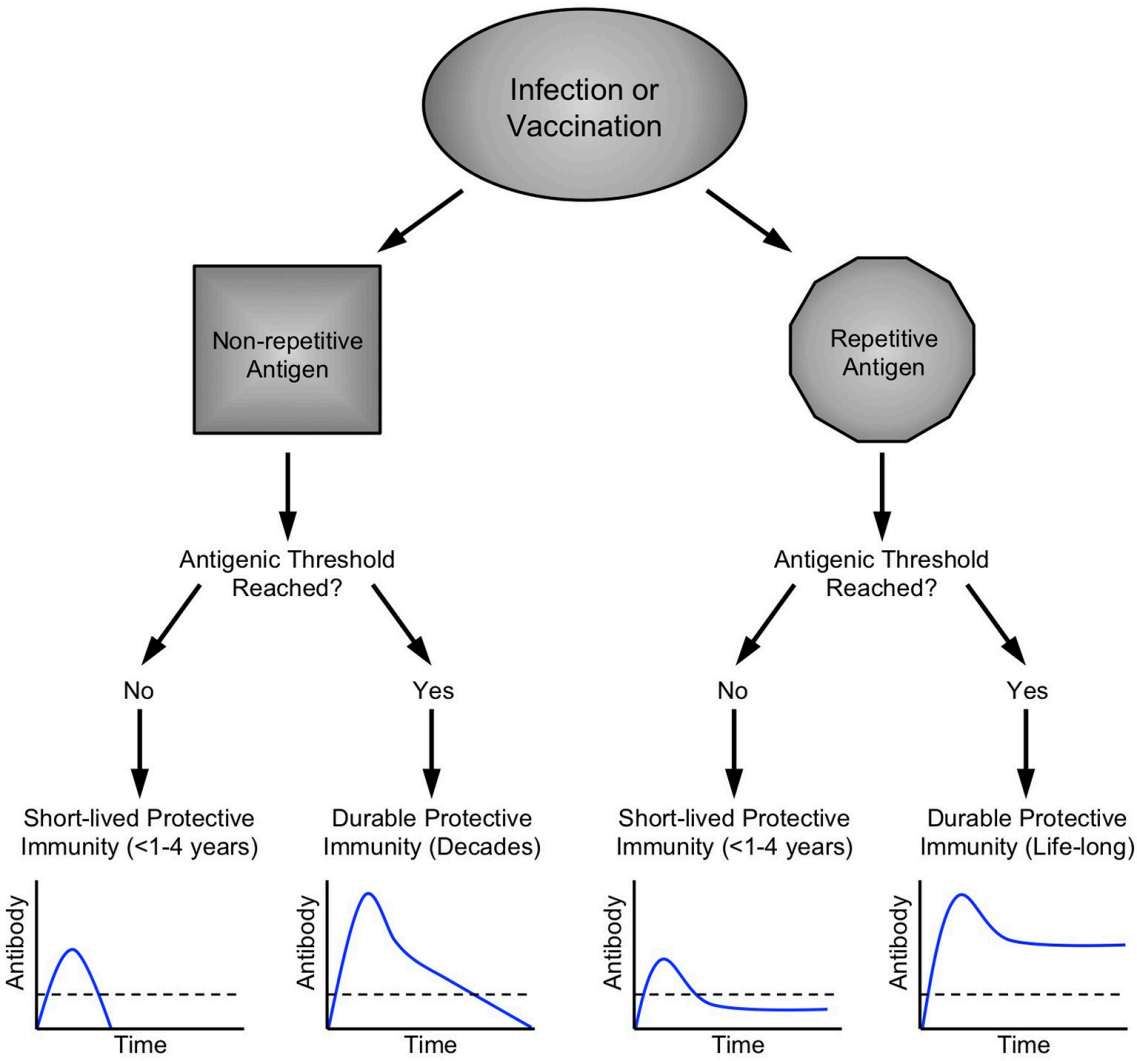
Imprinted lifespan model of humoral immunity. Prior longitudinal studies have indicated that different vaccine and virus antigens elicit serum antibody responses of varying duration (58). Since long-lived PC are terminally-differentiated non-dividing cells that can survive for months or even years after the initial antigenic insult has been resolved (27), our hypothesis is that PC lifespan must be determined or “imprinted” at the time of B cell activation during the initial stages of an active immune response. Microbes such as viruses and bacteria have surface antigens that are highly repetitive and these structural attributes are likely to cause increased B cell activation by binding to multiple immunoglobulin molecules on the responsive B cell and trigger increased signal transduction in comparison to B cell activation induced by non-repetitive or monovalent antigens. B cell activation is further enhanced via T cell help during T-dependent immune responses to foreign antigens. In addition to the structural attributes of a particular antigen, another important parameter is antigenic load (118). If an infection is resolved too quickly or if a vaccine has low antigen content, is rapidly degraded, or contains no adjuvant, then there will be fewer germinal center reactions, fewer activated B cells and T cells, and fewer PC that enter the long-lived pool. Bearing this in mind, we postulate that a certain antigenic threshold must be met in order to elicit a sufficient number of PC to maintain antibody responses above a protective threshold (shown as a dashed line in each graph) and this set point will differ depending on the level of protection needed for each pathogen or toxin. Contemporary detergent-disrupted influenza vaccines provide an example of non-adjuvanted non-repetitive antigen that does not reach the antigenic threshold. Under these circumstances, transient, low levels of immunity rapidly wane to below a protective threshold, often before the end of single influenza season. In contrast, booster vaccination against other non-repetitive alum-formulated antigens such as tetanus or diphtheria is able to elicit high antibody titers that decline faster than that observed with highly repetitive viral antigens (58), but nevertheless provide long-term immunity that may last for decades. Among repetitive antigens, the duration of immunity is also likely to be influenced by antigenic threshold. For highly attenuated vaccine strains of viruses such as MVA or Dengvaxia (Table 1), viral antigen may be cleared too quickly, resulting in the induction of fewer long-lived PC due to insufficient antigen load with an end-result of long-term antibody production that resides near or below the protective threshold. On the other hand, natural infections with viruses such as measles, vaccinia, influenza, etc., (Table 1) or immunization with optimized and adjuvanted VLP or whole-virus vaccines (Figures 1, 2) will potentially induce lifelong immunity by presenting repetitive epitopes while also achieving sufficient antigenic load that, together with appropriate T cell help, will result in long-term antibody production that is maintained at or above protective levels.

The impact that antigen structure (i.e. monomeric, multimeric, aggregated) can have on antigen processing, cellular immunity and subsequent B cell responses has been well described (96–99). Many of the concepts regarding the role of B cell epitope multivalency, antigenic thresholds, and the Imprinted Lifespan model for generating long-lived PC (Figure 4) have been previously described (14, 38). According to the Imprinted Lifespan Model, exposure to haptens or monovalent self antigens are most likely to elicit only short-term immunity due to the lack of B cell receptor crosslinking/activation and the absence of CD4^+^ T cell help. Vaccines containing monomeric foreign protein antigens such as tetanus or diphtheria do not induce substantial B cell receptor crosslinking but due to peptide-specific CD4^+^ T cell help, these antigens are immunogenic and induce protective immune responses that can last for many years. Vaccines containing multimeric foreign protein antigens would be expected to elicit the highest level of B cell receptor cross-linking that, in addition to cognate CD4^+^ T cell help, will “imprint” the activated B cells to proliferate and differentiate into plasma cell populations that are more long-lived than those elicited by monovalent antigens and may in some circumstances result in life-long immunity.

The concept that all viral infections are expected to elicit lifelong immunity stems mainly from observations of the durable immunity induced by natural infection. Live-attenuated vaccines have been developed to mimic the protective immune responses elicited by natural infection but without prolonged and/or high-titer infection causing the clinical manifestations of that particular disease. The steps taken to ensure attenuation and increased safety often comes at the cost of reduced immunogenicity due to lower antigen load and this associated reduction in antigenic threshold may result in short-lived or only partial protective immunity in the absence of at least one booster vaccination (Table 1). Although natural infection with dengue virus (DENV) may not provide complete protection against reinfection (100), it is generally believed to provide long-term immunity since the majority of DENV cases and the more severe disease is identified more often among children than adults, indicating that adults living in endemic communities are afforded serotype-specific protection presumably from prior childhood infection (101). Based on this premise, it was expected that a live-attenuated dengue virus vaccine would likewise induce durable protective immunity, but recent studies indicate that this was not the case. Following 3 doses of the live-attenuated Dengvaxia vaccine (comprised of a chimeric yellow fever virus expressing dengue serotype-specific envelope proteins), children 2–5 years of age in the ASIA (CYD14) longitudinal cohort showed ~36% VE (Vaccine Efficacy = 1–hazard ratio) during the first year after vaccination, but no vaccine-mediated protection remained by 3 years post-vaccination (102). Likewise, children among cohorts aged 6–8 years or 9–11 years of age showed 79% VE and 50% VE during the first year after vaccination but protection dropped to undetectable levels by 4 years and 3 years post-vaccination, respectively. Dengvaxia VE was not statistically significant during the first year after vaccination for two of the three age groups and it is notable that even trends toward vaccine-mediated protective immunity were subsequently lost within 3–4 years after a 3-dose vaccination series (102). Moreover, lack of vaccine efficacy and concerns with potential vaccine-associated exacerbation of disease among previously seronegative subjects (103) have resulted in the Dengvaxia vaccine indication being changed to include only dengue-seropositive individuals.

**Table 1:** Examples of antiviral immunity generated from wild-type viruses vs. live-attenuated vaccine strains.

**Virus or vaccine target**	**Repetitive structure?**	Reach antigenic threshold?^*^	**Years**	**Duration of Immunity Decades**	**Lifelong**
Dengue	Yes	Yes	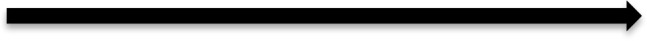		
Dengvaxia	Yes	No	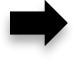		
Measles	Yes	Yes	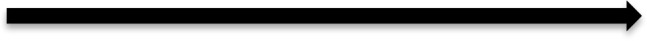		
Measles (MMR)	Yes	Partial	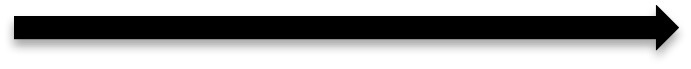		
Mumps	Yes	Yes	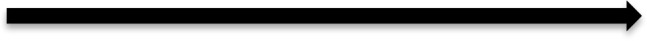		
Mumps (MMR)	Yes	Partial	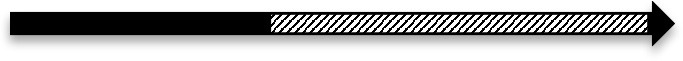		
Vaccinia-Dryvax	Yes	Yes	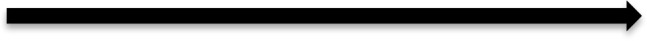		
Vaccinia-MVA	Yes	No	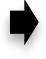		
Varicella zoster virus	Yes	Yes	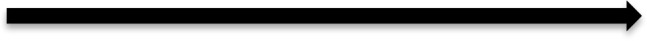		
VZV-Oka	Yes	Partial	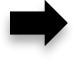		
Yellow fever	Yes	Yes	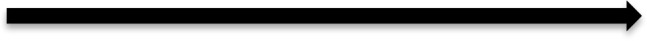		
YFV-17D/17DD	Yes	Partial	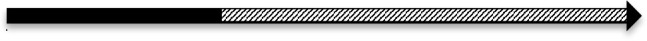		

* *Antigenic Threshold is defined as follows: Yes: indicates that the infection achieves a high level of antigenic stimulation that induces antigen-specific B cells to proliferate and differentiate into a sufficient number of PC to provide long-term antibody production above a protective threshold. Partial: a partial achievement of antigenic threshold is defined as a smaller degree of antigenic stimulation resulting in shorter duration of immunity that may decline below the protective threshold but can be improved by booster vaccination. No: If there is little or no achievement in reaching an antigenic threshold then this means that even after booster vaccination, antiviral immunity will decline to below protective levels in the majority of individuals in < 1–4 years. Cross-hatched arrows indicate that immunity may be long-lived in a subpopulation of individuals. MMR, measles, mumps, rubella; MVA, modified vaccinia Ankara; VZV, varicella zoster virus*.

It is well-established that childhood measles infection induces lifelong immunity (104), with serum antibody responses that show little or no decay when measured longitudinally for over 20 years (58). In contrast, after achieving 95–99% seroconversion to measles after primary MMR (measles, mumps, rubella) vaccination (105), there is evidence suggesting that immunity may wane (106) and a 2-dose schedule is recommended in order to raise the antigenic threshold and induce antibody titers that plateau above the protective threshold. Likewise, when we examined the durability of measles-specific antibodies among a small group of MMR vaccine recipients, the antiviral antibody levels were stable but in three out of four subjects, the titers were maintained at or below the protective threshold of 1,000 ELISA Units/mL, equivalent to 0.2 IU/mL [(58); Supplemental Appendix] whereas the majority of antibody responses to natural measles infection were maintained at much higher levels, most likely due to the prolonged infection and exposure to higher antigenic load during natural infection vs. vaccination (38, 58). Similar to measles, mumps is another common childhood infection that typically results in life-long immunity. However, recent studies have indicated that there may be a greater waning of MMR vaccine-mediated immunity against mumps then that observed against measles and the ACIP currently recommends a 3rd dose of MMR vaccine be administered to persons previously vaccinated with 2 doses who are at increased risk for contracting mumps during an outbreak (107).

Although smallpox has been eradicated, studies involving different smallpox vaccines have been informative in understanding long-term antiviral immunity. Vaccinia virus strains used in standard scarification/transcutaneous smallpox vaccination have not been specifically attenuated for vaccine use and they elicit long-term antibody and T cell-mediated immunity similar to Variola virus itself (108). In contrast, the MVA (modified vaccinia Ankara) strain of vaccinia virus was heavily attenuated by serial *in vitro* passage in chicken embryo fibroblast cells, resulting in a virus that is essentially replication-deficient in humans. Unlike replicating strains of vaccinia virus that induce high seroconversion rates (>90%) and long-term immunity after a single vaccination [antibody T_1/2_ = 92 years (58)], immunization with 2 doses of MVA result in ~71% seroconversion for neutralizing antibodies (≥1:10) at the peak of the immune response before declining to 28.8% seropositive status within 2 months after booster vaccination (109). Another study provided similar results; within 6 months after booster MVA vaccination, antibody titers waned to near baseline levels and seroconversion rates declined to 35–54%, depending on the formulation and immunization route (110).

Chickenpox is caused by varicella zoster virus (VZV) and similar to measles and mumps, it was considered to be a childhood disease because after recovery from primary infection, it was rare to get chickenpox twice. VZV is an α-herpesvirus that establishes latency within the infected host and therefore it was believed that this vaccine would require only one shot to elicit lifelong immunity. Instead, although high levels of protection are observed during the first 1–3 years after primary vaccination, protective immunity continuously wanes thereafter, resulting in a >30-fold increase in the risk of symptomatic breakthrough cases by 9 years after vaccination (111). In 2006, the ACIP recommended a second dose of varicella vaccine be administered to improve long-term vaccine-mediated immunity.

Yellow fever virus (YFV) causes an acute viral infection and similar to other acute viral infections (dengue, measles, mumps, vaccinia, etc.) recovery from natural YFV infection induces essentially lifelong immunity (112). Development of live-attenuated YFV vaccines are believed to also induce lifelong immunity and primary seroconversion rates of >95% have been noted in multiple clinical trials (113). However, despite initially high rates of seroconversion and long-term immunity among the majority of vaccinated subjects, it appears that 20–30% of YFV-immunized adults may lose immunity within the first 5–10 years after primary vaccination (113–115). Bearing in mind that initial seroconversion rates are substantially lower among children [69–85% primary seroconversion (116, 117)], there is concern that subsequent waning immunity might result in an even larger number of children/adolescents who become unprotected or seronegative if a booster dose is not administered. For these reasons, some YFV-endemic countries such as Brazil have continued to maintain booster vaccinations against yellow fever.

## The Imprinted Lifespan Model

In 2007, we published a longitudinal study in which we found a surprising difference in the durability of serum antibody responses mounted against monovalent protein antigens such as tetanus and diphtheria toxins vs. the persistence of antibody responses to multivalent antigens associated with measles, mumps, rubella, or vaccinia infection (58). Based on these observations, we proposed the Imprinted Lifespan Model of long-lived PC (14, 38), wherein PCs are imprinted with a predetermined lifespan based on the magnitude and quality of B cell signaling that occurs during the induction of an antigen-specific humoral immune response (Figure 4). This model is based primarily on a set of 4 core tenets. Tenet #1: Long-lived PC are defined as terminally differentiated non-dividing cells that continuously secrete antibodies without requiring further stimulation. Since most PC lose the expression of surface-bound immunoglobulins and down-regulate MHC Class II expression, they are less likely to sense their antigenic microenvironment and will have little or no direct antigen-specific interactions with CD4^+^ T cells. Bearing this in mind, we propose that the intrinsic lifespan of these non-dividing cells must be determined during the induction of the humoral immune response when the responding B cells first encounter their cognate antigen. Tenet #2: The majority of long-lived PC reside in the bone marrow and since there is only finite space within the bone marrow compartment for new PC, it is possible that resident PC are imprinted with a lifespan of varying duration based on the probability that the antigen represents a relevant and protective target epitope. Antibody responses to soluble self antigens or carbohydrates are normally avoided due to the monomeric nature of the soluble antigen and the lack of T cell help. Highly repetitive/multimeric carbohydrate antigens may be recognized as being from an invading microbe based on their multivalent structures and although they are capable of eliciting T cell-independent antibody responses by virtue of their ability to activate B cells through crosslinking of the B cell receptor (BCR), these types of antibody responses are often short-lived unless the antigen is conjugated to a carrier protein in order to allow the secondary signals to be initiated through critical B cell:T cell interactions (14, 38, 119). Tenet #3: Foreign protein antigens capable of triggering the activation of B cells can be divided into two general categories; monovalent and multivalent. The surface structures on particulate antigens such as viruses and bacteria are often highly repetitive (i.e., multivalent) whereas internal proteins are more likely to be monomeric (e.g., non-structural proteins, viral or bacterial RNA/DNA polymerases, etc.), the induction of PC with a preferentially longer lifespan against highly repetitive antigens could provide a long-term advantage to the host by not crowding the bone marrow with PC to potentially less protective epitopes among internal microbial proteins. However, even monomeric foreign proteins can be dangerous to the host and therefore antibody responses to secreted virulence proteins or toxins (tetanus toxin, diphtheria toxin, pertussis toxin, etc.) is also needed, but since these proteins lack the repetitive nature of microbial surface antigens, the PC responses would be imprinted with an intermediate lifespan that may last for years or decades rather than being dedicated to nearly a lifetime of antibody production in the bone marrow. Tenet #4: In order to achieve long-term protective immunity, an antigenic threshold must be reached in order to generate sufficient B cell activation, proliferation, and differentiation into a minimum number of PC to maintain antibody production at levels above a protective threshold for that particular pathogen or toxin.

Exposure to a non-repetitive foreign antigen during infection or vaccination will trigger initial B cell activation and antibody production (Figure 4). If this is a primary exposure/vaccination then it is possible that the antigenic threshold is not reached and antibody responses will peak and then decline below protective levels shortly thereafter. Influenza vaccination with detergent-disrupted “split” virus falls into this category, with only short-lived immunity that may or may not last for a single influenza season (Figure 3A). However, if an appropriate adjuvant is included [e.g., alum (120, 121)] to create an antigen depot and/or if booster vaccination is performed, then the peak immune response is likely to be higher and after the early stages of rapid antibody decline (14) (Figures 1, 2, and 3), it is anticipated that antibody levels will decay at a slow rate that is potentially maintained for years above the protective threshold. Antibody responses to tetanus and diphtheria toxins fall into this category. After the initial rapid antibody decay that occurs for 1–3 years post-vaccination, anti-toxin antibody responses decline with an estimated half-life of 11–14 years (58, 69, 122) for tetanus and 19–27 years (58, 122) for diphtheria, respectively. Interestingly, diphtheria-specific antibody decay rates observed among people naturally exposed to endemic diphtheria are higher than those vaccinated after elimination of indigenous diphtheria but the antibody decay rate kinetics appear to be essentially the same (123). This suggests that the antibody decay rates for monomeric antigens will be similar regardless of whether the exposure comes from an infection or through vaccination. Although antitoxin antibody responses are likely to continue declining over time, their initial peak levels are sufficiently above the protective threshold to allow protective immunity to persist for many years. Notably, the World Health Organization does not recommend adult booster vaccination against tetanus or diphtheria once the initial 5-dose vaccination series has been completed (124, 125). Likewise, we have examined the incidence of tetanus and diphtheria among countries that do or do not routinely vaccinate adults and found that continued vaccination of the adult population may no longer be needed once the childhood vaccination series has been completed (A. Slifka, L. Gao, M. Slifka; manuscript submitted). This indicates that even monomeric antigens can elicit lifelong immunity if booster vaccination reaches a minimum antigenic load resulting in antibody titers that are sufficiently higher than what is needed to be maintained above a protective threshold.

Following infection or vaccination with highly repetitive antigens, protective immunity can be short-lived or long-lived depending on the antigenic threshold. Following infection/vaccination with highly attenuated live viruses such as MVA (109, 110) or Dengvaxia (102), antiviral immunity may crest above the protective threshold but decline to levels that reside below those needed for long-term immunity, presumably because the viral antigens are cleared too rapidly to elicit a large PC population that can survive and maintain antibodies above protective levels. In contrast, when highly repetitive antigens are expressed during natural infection such as measles, mumps, etc. (Table 1) or when immune responses are elicited through booster vaccination with live-attenuated viruses or immunization with highly repetitive whole-virus or VLP antigens formulated with an appropriate adjuvant (Figures 1, 2), then long-term antibody responses can be generated that plateau above the protective threshold during the maintenance phase and result in potentially life-long immunity.

Understanding the underlying mechanisms for inducing lifelong antibody responses is key to rational vaccine design. Natural influenza infection results in lifelong subtype-specific antiviral immunity whereas current detergent-disrupted influenza vaccines often fail to protect against disease for even a single season (Figure 3A) even in the absence of appreciable antigenic drift (83, 91). The poor immunogenicity of detergent-split influenza vaccines has been demonstrated in head-to-head comparisons with whole-virus vaccines (Figure 3B) and with the contemporary lack of high vaccine efficacy resulting in large scale hospitalizations and nearly 80,000 deaths in the US during the 2017/2018 season (77), it is time that we re-evaluate the design of future vaccines with an emphasis on the structural integrity of vaccine antigens. It is possible that even small but fundamental changes in vaccine design could improve early antiviral immune responses (Figure 3B) as well as lead to more durable antibody responses that last beyond a single flu season (Figure 3C).

The role of germinal center reactions including interactions with different CD4^+^ T cell subsets, adhesion molecules, cytokines, chemokines, metabolic pathways, and many other aspects of the immediate microenvironment encountered during both initial B cell stimulation as well as PC homing and survival in the bone marrow microenvironment are all important areas that are worthy of further investigation. More studies are needed to better understand the dynamics of B cell activation during vaccination or infection and we believe that optimization of these parameters, especially with regard to antigenic threshold (to determine antibody magnitude) and antigen structure (to determine antibody persistence), will lead to improved vaccines in the future that elicit more durable immunity not only against influenza but for other major human pathogens as well.

## Author Contributions

MS and IA contributed to manuscript drafting and revision. Both authors read and approved the submitted version

### Conflict of Interest Statement

IA is the Vice President for Research at Najít Technologies, Inc. MS is a Professor at Oregon Health & Science University and the President and Chief Scientific Officer at Najít Technologies, Inc.
